# *Drosophila insulin receptor* regulates diabetes-induced mechanical nociceptive hypersensitivity

**DOI:** 10.17912/micropub.biology.000456

**Published:** 2021-09-16

**Authors:** Harika Dabbara, Arielle Schultz, Seol Hee Im

**Affiliations:** 1 Department of Biology, Haverford College, Haverford, PA

## Abstract

Painful diabetic neuropathy (PDN) is one of the predominant complications of diabetes that causes numbness, tingling, and extreme pain sensitivity. Understanding the mechanisms of PDN pathogenesis is important for patient treatments. Here we report *Drosophila* models of diabetes-induced mechanical nociceptive hypersensitivity. Type 2 diabetes-like conditions and loss of insulin receptor function in multidendritic sensory neurons lead to mechanical nociceptive hypersensitivity. Furthermore, we also found that restoring insulin signaling in multidendritic sensory neurons can block diabetes-induced mechanical nociceptive hypersensitivity. Our work highlights the critical role of insulin signaling in nociceptive sensory neurons in the regulation of diabetes-induced nociceptive hypersensitivities.

**Figure 1. Type 2 diabetes-like conditions and loss of insulin receptor function cause mechanical nociceptive hypersensitivity in Drosophila larvae:**
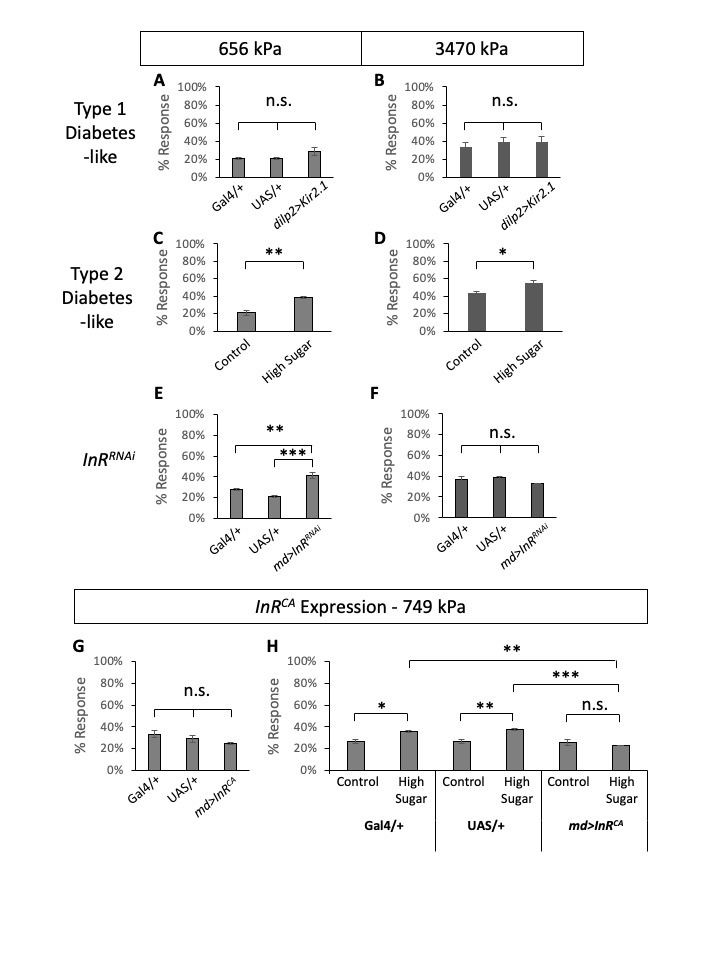
Average percent responses to noxious mechanical stimuli of 3^rd^ instar larvae with 656 kPa probe (A, C, E), 749 kPa probe (G, H), and 3470 kPa probe (B, D, F). Error bars represent S.E.M. (A-B) Larvae with Type 1 diabetes-like condition showed no significant differences compared to the two controls for neither 656 kPa nor 3470 kPa probes. Type 1 diabetes model of *dilp2>Kir2.1* larvae was compared to the two controls, *dilp2-Gal4* alone and *UAS*–*Kir2.1* alone. (C-D) Type 2 diabetes model of high sugar diet-fed larvae showed increased mechanical nociceptive sensitivity compared to control larvae (control-diet fed) with significant differences for both 656 kPa and 3470 kPa probes. (E-F) *Insulin receptor* (*InR*) knockdown in multidendritic neurons using *md-Gal4*. *InR* knockdown caused increased mechanical nociceptive sensitivity compared to the two controls for the 656 kPa probe but not for the 3470 kPa probe. (G-H) Expression of the constitutive form of InR (*InR^CA^*) in multidendritic neurons was tested with a 749 kPa mechanical pressure probe. (G) Expression of *InR^CA^* did not alter mechanical nociception in response to the 749 kPa probe when cultured on a regular cornmeal fly food. (H) Larvae with *InR^CA^* expression did not show altered mechanical nociception while the two controls showed hypersensitivity induced by a type 2 diabetes-like condition in response to the 749 kPa probe. Under high sugar diet conditions, larvae with *InR^CA^* expression showed reduced mechanical nociceptive responses with significant differences compared to the controls. A-E, G-H: n = 30 triplicates tested, F: n = 30 duplicates tested. One-way ANOVA with Tukey’s post-hoc test (A-B, E-H) and T-test (C-D) were performed to test statistical significance (*, *p<0.05;* **, p <0.01; ***, p<0.001; n.s., not significant).

## Description

Diabetes is a group of medical conditions that involve the dysregulation of blood glucose and is typically categorized into two types. Type 1 diabetes is an autoimmune disease that results from the immune system attacking insulin-producing cells in the pancreas, causing hyperglycemia and hypoinsulinemia (American Diabetes Association 2014). Type 2 diabetes is characterized by insulin receptors on the membranes of cells becoming resistant to insulin, leading to hyperglycemia and hyperinsulinemia (Petersen and Shulman 2018). Both types of diabetes can trigger complications called painful diabetic neuropathy (PDN). Patients with PDN suffer from a range of symptoms including numbness and tingling in distal extremities and extreme sensitivity to stimuli, such that the touch of one’s clothing on the skin can be painful (Veves *et al.* 2008). To improve treatment options for these patients, it is important to develop a genetically tractable model system to dissect the underlying mechanisms and test new therapeutics. Previously, we reported a *Drosophila* model of diabetes-induced nociceptive hypersensitivity (Im *et al.* 2018). In that study, we established that diabetes-like conditions and disrupted insulin signaling result in prolonged thermal nociceptive hypersensitivity after tissue injury.

Here, to address clinically relevant mechanical pain hypersensitivity, we tested *Drosophila* models of diabetes for their mechanical nociceptive sensitivity using a recently improved *Drosophila* mechanical nociception assay (Lopez-Bellido *et al.* 2019, Lopez-Bellido and Galko 2020). We found that larvae with type 2 diabetes-like conditions exhibit mechanical nociceptive hypersensitivity. Similarly, loss of *insulin receptor* function in multidendritic sensory neurons resulted in mechanical nociceptive hypersensitivity. We also found that constitutively activating insulin signaling in multidendritic sensory neurons can restore normal mechanical nociceptive sensitivity in type 2 diabetic larvae. Taken together, our results demonstrate the usefulness of our genetically tractable model system for dissecting and studying molecular and cellular mechanisms of diabetes-induced mechanical nociceptive hypersensitivity and emphasize the critical role of insulin signaling in sensory neurons to prevent diabetes-induced nociceptive hypersensitivity.

First, to test if diabetes alters mechanical nociceptive sensitivities in *Drosophila* larvae, we tested both type 1 and type 2 diabetes models of *Drosophila* larvae. To model type 1 diabetes, we expressed inward rectifying potassium channels (*Kir2.1*) in the insulin-producing cells (IPCs) using *dilp2-Gal4* and blocked the release of Dilps from IPCs (Kim and Rulifson 2004). We tested type 1 diabetes-like, and *Gal4*– and *UAS*-alone control larvae in mechanical nociception assays (n=30 triplicates) using two different pressure mechanical probes, one conveying 656 kPa and the other 3470 kPa. When tested in wildtype *Canton S* larvae, 656 kPa pressure resulted in ~20% response rate while 3470 kPa pressure resulted in ~40% response rate, similar to what was reported previously (Lopez-Bellido *et al.* 2019). Larvae in type 1 diabetes-like condition did not show any significant changes in mechanical nociceptive sensitivity (Figure 1 A-B). We modeled Type 2 diabetes by feeding larvae a high sugar diet similar to previous studies (Palanker Musselman *et al.* 2011, Im *et al.* 2018). When compared to control diet-fed larvae, high sugar diet-fed larvae (type 2 diabetes-like) showed increased nociceptive sensitivities in response to both pressure mechanical probes (n=30 triplicates, Figure 1 C-D). The differences between the control and type 2 diabetes-like larvae were statistically significant. This suggests that similar to humans, diabetes-like fly larvae experience diabetes-induced mechanical nociceptive hypersensitivity (Veves *et al.* 2008).

Next, we tested the role of *insulin receptor* (*InR*) in mechanical nociception. Previously, we examined the effect of *InR* knockdown in multidendritic sensory neurons for thermal nociceptive sensitization and found that *InR* knockdown causes prolonged thermal nociceptive hypersensitivity after injury (Im *et al.* 2018). Similarly, here we tested RNAi-mediated knockdown of *InR* in multidendritic sensory neurons using a pan-multidendritic sensory neuron driver, *md-Gal4* (*Gal4^109(2)80^*) (Gao *et al.* 1999), and monitored mechanical nociceptive sensitivity. Previously, *md-Gal4*-driven *InR*knockdown was tested and confirmed by another pan-multidendritic sensory neuron driver (*21-7-Gal4*) (Im *et al.* 2018). Compared to the *Gal4* alone and *UAS* alone controls, *InR^RNAi^*-expressing larvae showed an increase of sensitivity to the low-pressure probe (656 kPa) (Figure 1E). In comparison, the responses to a higher pressure 3470 kPa mechanical probe were not significantly different among the experimental and the control genotypes (Figure 1F). This suggests that hypersensitivity to low-pressure mechanical stimuli observed in type 2 diabetic larvae can be explained by the loss of insulin receptor function in multidendritic sensory neurons, while the observed hypersensitivity to high-pressure stimuli might be more complicated.

Lastly, we tested if restoring insulin signaling in multidendritic sensory neurons could rescue diabetes-induced mechanical nociceptive hypersensitivity. To constitutively activate insulin signaling, we expressed a constitutively active form of *InR* (*InR^CA^*) using the *Gal4/UAS* system (Wang *et al.* 2008). Expression of *InR^CA^* under normal diet conditions did not alter mechanical nociception (Figure 1G), suggesting that activating the insulin receptor in non-diabetic larvae does not change mechanical nociceptive sensitivity. Next, we tested the control diet and high-sugar diet conditions of the *GAL4*– and *UAS*-alone controls and *InR^CA^*-expressing larvae for their mechanical nociception using a low-pressure mechanical probe (749 kPa) (Figure 1H). The control genotypes (*Gal4-* and *UAS-*alone) exhibited increased mechanical nociceptive sensitivities in the high sugar diet compared to the matching genotype under the control diet, demonstrating the same hypersensitivity observed in wildtype *Canton S* larvae under the high sugar diet (Figure 1C-D). In contrast, *InR^CA^*-expressing larvae showed comparable sensitivities in both the control diet and the high sugar diet (Figure 1H). Statistical analysis showed that there is no significant difference between the control and the high sugar diet mechanical nociceptive responses of *InR^CA^*-expressing larvae. This result shows that activation of insulin signaling in multidendritic sensory neurons can block diabetes-induced mechanical nociceptive hypersensitivity.

This study demonstrates the critical role of insulin signaling in diabetes-induced mechanical nociceptive hypersensitivity. We found that loss of insulin signaling, either due to diabetic conditions or genetic manipulation of the insulin receptor expression, causes mechanical nociceptive hypersensitivity. We also found that this hypersensitivity can be rescued by constitutively activating insulin signaling in multidendritic sensory neurons. The cause of diabetes-induced pain is widely debated, as diabetes has many associated changes in physiological conditions (blood sugar levels, local ischemia, tissue damage due to imbalanced nutrients, undesirable byproduct accumulation, etc.) (Obrosova 2009, Zochodne 2016). Our results highlight one important factor for PDN development to consider – the systemic loss of insulin signaling. This loss occurs early in diabetic conditions and nociceptive sensory neurons themselves require active insulin signaling to maintain their normal functions (Vincent *et al.* 2011, Grote and Wright 2016).

It is worthwhile to compare two different pain modalities – thermal nociception (Im *et al.* 2018) and mechanical nociception (current study). It is intriguing that we observed different kinetics between these two pain modalities. For thermal nociception, diabetes-like conditions do not alter baseline thermal nociception sensitivity as diabetic larvae and controls are not different in their responsiveness (Im *et al.* 2018). It was during the recovery state after injury when we observed drastic differences between diabetes-like and non-diabetic controls in thermal nociception – non-diabetic controls recover normally from injury-induced nociceptive sensitization while diabetes-like larvae (and loss of insulin receptor function) did not recover and exhibit prolonged hypersensitivity after injury (Im *et al.* 2018). Thus, diabetes-like larvae have no problem with thermal pain modality until they are exposed to tissue damage. For mechanical nociception, we detect hypersensitivity in uninjured larvae in type 2 diabetes-like conditions (Figure 1C-D). This suggests that the type 2 diabetic conditions affect mechanical nociceptive sensitivity right away without tissue injury modulating cellular signaling (Babcock *et al.* 2009, Babcock *et al.* 2011, Im *et al.* 2015, Jo *et al.* 2017). What might be the mechanisms that drive the varying kinetics of nociceptive hypersensitivity between these two pain modalities? It might be the regulation of different molecular receptors involved in distinct pain modalities (Tracey *et al.* 2003, Zhong *et al.* 2010, Gorczyca *et al.* 2014, Guo *et al.* 2014).

Our findings also highlight differences between type 1 and type 2 in diabetes-induced mechanical nociceptive hypersensitivity. Type 1 diabetes-like condition did not affect mechanical nociception sensitivity in response to neither low- or high-pressure mechanical probes, while Type 2 diabetes-like condition showed hypersensitivities in response to both low- and high-pressure probes (Figure 1A-D). This indicates that type 2 diabetes-like conditions might be more prone to developing mechanical nociceptive hypersensitivity compared to type 1 diabetes-like conditions. This is another interesting difference between nociceptive modalities as we pointed out previously. For the prolonged thermal nociceptive hypersensitivity, both type 1 and type 2 diabetes-like conditions display the same phenotypes, but we observe differences between type 1 and type 2 diabetes-like conditions in mechanical nociceptive hypersensitivity. Putting these findings together, it might indicate that types 1 and 2 diabetes have different underlying mechanisms for PDN pathogenesis similar to their different pathogenesis mechanisms of diabetes. Using our diabetes-induced pain models, it will be exciting to further dissect the molecular and cellular mechanisms of PDN pathogenesis and develop better treatments to alleviate patients’ pain symptoms.

## Methods

*Fly stocks and fly food* – Stocks were obtained from the Bloomington *Drosophila* Stock Center and cultured on a regular cornmeal media (10 g/L agar, 27.5 g/L brewer’s yeast, 52 g/L cornmeal, 11 g/L sucrose, 4.5 mL/L propionic acid, 0.1% mold inhibitor) except for the type 2 diabetes experiments. All experimental crosses were reared at 25 °C. A high-sugar diet (10 g/L agar, 80 g/L brewer’s yeast, 20 g/L yeast extract, 20 g/L peptone, 342 g/L sucrose, 0.5 g/L MgSO_4_, 0.5 g/L CaCl_2_, 6 ml/L propionic acid, 0.1% mold inhibitor) contains 6.7 times higher sugar compared with a control diet (51 g sucrose, all other ingredients the same) (Palanker Musselman *et al.* 2011). Multidendritic sensory neuron-specific expression of UAS transgenes was controlled by *Gal4109(2)80* (Gao *et al.* 1999); insulin-producing cells (IPCs) by *dilp2-Gal4* (Rulifson 2002). *UAS-Kir2.1* was used to silence IPCs and block dilp secretion (Kim and Rulifson 2004); *UAS- InR^JF01482^* (*InR^RNAi^*) (Dietzl *et al.* 2007, Ni *et al.* 2011) and *UAS-InR*^A1325D^ (*InR^CA^*) (Wang *et al.* 2008) were used to manipulate *InR* function. *Gal4* alone and *UAS* alone controls were crossed to *w^1118^*.

*Larval mechanical nociception assay* – Mechanical nociception assays were performed as described previously (Lopez-Bellido *et al.* 2019, Lopez-Bellido and Galko 2020). Briefly, mechanical probes were built by gluing nitinol filaments to craft sticks and calibrated to apply a measured amount of pressure when they bend against a surface. The probes we used in this study carry 656 kPa, 749 kPa, and 3470 kPa. To monitor mechanical nociception behavior responses, each mechanical probe was applied to the posterior dorsal side of the larva (abdominal segment A8) and maintained bent configuration for 1-2 seconds. When the probe is lifted, a larva performing a 360-degree body roll was considered as a positive responder. Other responses, such as crawling, turning, or wiggling, were not counted as nociceptive responses. Each set tested 30 larvae per genotype/condition, and three independent replicate experiments were performed except Figure 1F, which were performed duplicate of n=30.

*Statistical Analysis Results*

**Table:** 

***Figure Panels***	***Statistical Test***		***P value***
*A*	*ANOVA*		*0.1363*
*B*	*ANOVA*		*0.7568*
*C*	*Unpaired t test*		*0.0048*
*D*	*Unpaired t test*		*0.0375*
*E*	*ANOVA*		*0.0008*
*E*	Tukey’s post-hoc test	*Gal4/+ vs. UAS/+*	*0.0886*
*E*	Tukey’s post-hoc test	*Gal4/+ vs. MD>InR[RNAi]*	*0.006*
*E*	Tukey’s post-hoc test	*UAS/+ vs. MD>InR[RNAi]*	*0.0007*
*F*	*ANOVA*		*0.336*
*G*	*ANOVA*		*0.1366*
*H*	*ANOVA*		*0.0003*
*H*	Tukey’s post-hoc test	*Gal4/+: Cont vs. HS*	*0.0337*
*H*	Tukey’s post-hoc test	*UAS/+: Cont vs. HS*	*0.0068*
*H*	Tukey’s post-hoc test	*md>InR[CA] : Cont vs. HS*	*0.9879*
*H*	Tukey’s post-hoc test	*Cont : Gal4/+ vs. UAS/+*	*>0.9999*
*H*	Tukey’s post-hoc test	*Cont: Gal4/+ vs. md>InR[CA]*	*>0.9999*
*H*	Tukey’s post-hoc test	*Cont : UAS/+ vs. md>InR[CA]*	*>0.9999*
*H*	Tukey’s post-hoc test	*HS: Gal4/+ vs. UAS/+*	*0.9881*
*H*	Tukey’s post-hoc test	*HS: Gal4/+ vs. md>InR[CA]*	*0.0032*
*H*	Tukey’s post-hoc test	*HS: UAS/+ vs. md>InR[CA]*	*0.0007*

## Reagents

*Drosophila melanogaster stocks used:*

**Table:** 

	Genotype	Source	Identifier
*md-Gal4*	y^1^ w^*^; P{GawB}^109(2)80^	Bloomington *Drosophila* Stock Center	FBst0008769
*dilp2-Gal4*	w*; P{Ilp2-GAL4.R}2/CyO	Bloomington *Drosophila* Stock Center	FBst0037516
*UAS-InR^RNAi^*	*y^1^ v^1^; P{TRiP.JF01482}attP2*	Bloomington *Drosophila* Stock Center	FBst0031037
*UAS-Kir2.1*	w*; UAS-Kir2.1	Gift from Kartik Venkatachalam	FBal0346841
*UAS-InR^CA^*	*y^1^ w^1118^; P{UAS-InR.A1325D}2*	Bloomington *Drosophila* Stock Center	FBst0008263
